# Editorial: Technostress, mental health and well-being

**DOI:** 10.3389/fpsyg.2023.1356844

**Published:** 2024-01-16

**Authors:** Barbara Masluk, Angela Asensio-Martínez, Silvia Lopes, Alejandro Vega-Muñoz

**Affiliations:** ^1^University of Zaragoza, Zaragoza, Spain; ^2^Aragonese Primary Care Research Group (GAIAP), Institute for Health Research Aragón (IIS Aragón), Zaragoza, Spain; ^3^Catholic University of Portugal, Lisbon, Portugal; ^4^Universidad Central de Chile, Santiago, Chile; ^5^Universidad Arturo Prat, Iquique, Chile

**Keywords:** technostress, mental health, well-being, outcomes, qualitative, quantitative, information overload

Technostress is a type of stress that results from the extended use of technologies (Tarafdar et al., 2017). During the COVID-19 pandemic, working-from-home arrangements increased due to confinement and lockdown measures. This Research Topic presents studies that examine information and communication technologies, focussing on stress and mental health. As both theoretical and practical studies are important within this field, the editorial team encouraged submissions that analyze the effects of different types of teaching carried out online and that also present outcomes on psychological wellbeing. The resulting Research Topic includes seven original studies from European countries as well as from China and Japan.

“*Information and communication technologies-assisted after-hours work: A systematic literature review and meta-analysis of the relationships with work–family/life management variables*” (Santos et al.).

The first investigation focuses on work-after-hours that is assisted by Information and Technology. Situations in which employees are contacted outside of work hours while also being in charge of children have led to the blurring of boundaries between personal and professional activities. This article presents a literature review on the topic and a meta-analysis of 37 available articles with work-related ICT use after-hours and work–family–life management variables. Interestingly, the authors offer an analysis of articles from before and after COVID-19, highlighting that ICT-assisted out-of-hours work was not only positively related to work-family-life enrichment but also to work-family-life conflict. It has also been found that neither gender nor the pre- or post-COVID period significantly affects the relationship between ICT-assisted out-of-hours work and work-family conflict.

“*Technostress at work during the COVID-19 lockdown phase (2020–2021): a systematic review of the literature*” (Bahamondes-Rosado et al.).

The second work is also a literature review focusing on the situations of severe confinement during the early pandemic, between 2020 and 2021. Using the keywords “COVID-19 work technostress,” the review aimed to provide a qualitative analysis of the technostressors caused by the COVID-19 pandemic. It investigates the main impacts of technostress on work caused by the COVID-19 pandemic and identifies the types of technostress that had the greatest impact on work during the COVID-19 pandemic period. The article explores: (1) The “relevance of technostress at work”; (2) The “impact of COVID-19 on remote work”; (3) “Workers and remote working”; and (4) “Contextual barriers to remote working.” The results demonstrate a relationship between work-related technostress during the confinement phase of the COVID-19 pandemic. Furthermore, stress related to technology, occupational and organizational conditions led to higher levels of technostress, with negative health-related outcomes.

“*High prevalence of anxiety, depression, and stress among remote learning students during the COVID-19 pandemic: Evidence from a meta-analysis*” (Xu and Wang).

The third article in the issue considers the mental health of distance university students. The article aimed to examine the prevalence of anxiety, depression, and stress among remote learning students during the COVID-19 pandemic through a meta-analysis of 36 original articles. The results indicated that the prevalence of anxiety and depression among higher education students was significantly higher than that of primary education students. It has also been found that medical and emergency medicine students suffered more from mental stress than their non-medical peers and traditional distance education equivalents.

“*Social support as a mediator in the relationship between technostress or academic stress and health: analysis by gender among university students*” (Asensio-Martínez et al.).

University students are also studied in the next article, which explores the role of social support as a mediator in the relationship between technostress or academic stress and health. This descriptive study was carried out by self-reported online survey in 2022. The main finding was that social support significantly and positively mediates the relationship between academic stress and self-perceived health in men. Results also indicated that women had higher levels of technostress and academic stress than men at the time.

“*Five major outcomes of digitalization: relevance of a survival personality type during COVID-19 pandemic*” (Hamamoto et al.).

The fifth article presents a model to explain adaptation to digitalized society, the first article to propose it (Hamamoto et al.). A two-phase study was conducted in which during the first phase a “digitalization outcome Inventory” and “digitalization background questionnaire” were created and in the second phase, models and investigating relationships between the outcomes and background factors were established.

“*Mindfulness and technostress in the workplace: a qualitative approach*” (Ioannou).

This Research Topic also includes an article on a therapeutic response to technostress based on the harmfulness of techno-stress and the benefits that come with practicing mindfulness techniques. Investigating the experiences of more mindful employees through semi-structured interviews enables learning from these practices, with potentially important implications for managers and practitioners who strive to improve employee well-being within organizations. The results offer some of the first in-depth insights into the relationship between mindfulness and technostress, finding a set of underlying strategies that the most conscious people implement in a situation of technostress.

“*A new scale to assess technostress levels in an Italian banking context: the Work-Related Technostress Questionnaire*” (Porcari et al.).

The last article in the Research Topic is a validation study of a Work-Related Technostress Questionnaire, which was used to assess technostress levels in an Italian banking context. The study presents the questionnaire (WRT-Q), undertaking an analysis of the role of gender and age in modulating manifestations of technostress. The second aim of this study was to compare subgroups (gender and age classes) on TS levels, using the newly validated instrument. The authors conclude by emphasizing that in the sample the average levels of technostress were relatively low, although some individual scores were high.

## Author contributions

BM: Writing—original draft, Writing—review & editing. AA-M: Writing—original draft, Writing—review & editing. SL: Writing—original draft, Writing—review & editing. AV-M: Writing—original draft, Writing—review & editing.
